# Non-pharmaceutical Interventions for Pandemic COVID-19: A Cross-Sectional Investigation of US General Public Beliefs, Attitudes, and Actions

**DOI:** 10.3389/fmed.2020.00384

**Published:** 2020-07-03

**Authors:** Bella Nichole Kantor, Jonathan Kantor

**Affiliations:** ^1^Harvard Extension School, Harvard University, Cambridge, MA, United States; ^2^Center for Global Health, University of Pennsylvania Perelman School of Medicine, Philadelphia, PA, United States; ^3^Center for Clinical Epidemiology and Biostatistics, University of Pennsylvania Perelman School of Medicine, Philadelphia, PA, United States; ^4^Department of Dermatology, University of Pennsylvania Perelman School of Medicine, Philadelphia, PA, United States; ^5^Florida Center for Dermatology, P.A., St Augustine, FL, United States

**Keywords:** COVID- 19, non-pharmaceutical interventions, SARS—CoV-2, quarantine, public attitudes

## Abstract

Non-pharmaceutical interventions (NPIs) represent the primary mitigation strategy for the COVID-19 pandemic. Despite this, many government agencies and members of the general public may be resistant to NPI adoption. We sought to understand public attitudes and beliefs regarding various NPIs and self-reported adoption of NPIs, and to explore associations between NPI performance and the baseline characteristics of respondents. We performed a cross-sectional age-, sex-, and race- stratified survey of the general US population. Of the 1,005 respondents, 37% (95% CI 34.0, 39.9) felt that NPIs were inconvenient, while only 0.9% (95% CI 0.3, 1.5) of respondents believed that NPIs would *not* reduce their personal risk of illness. Respondents were most uncertain regarding the efficacy of mask and eye protection use, with 30.6 and 22.1%, respectively, unsure whether their use would slow disease spread. On univariate logistic regression analyses, NPI adherence was associated with a belief that NPIs would reduce personal risk of developing COVID-19 [OR 3.06, 95% CI [1.25, 7.48], *p* = 0.014] and with a belief that NPIs were *not* difficult to perform [OR 1.79, 95% CI [1.38, 2.31], *p* < 0.0001]. Respondents were compliant with straightforward, familiar, and heavily-encouraged NPI recommendations such as hand-washing; more onerous approaches, such as avoiding face touching, disinfecting surfaces, and wearing masks or goggles, were performed less frequently. NPI non-adherence is associated with both outcome expectations (belief that NPIs are effective) and process expectations (belief that NPIs are not overly inconvenient); these findings have important implications for designing public health outreach efforts, where the feasibility, as well as the effectiveness, of NPIs should be stressed.

## Introduction

Non-pharmaceutical interventions (NPIs) have emerged as a first line of protection and mitigation in the face of the SARS-CoV-2 infection pandemic, particularly given the evidence suggesting the efficacy of such interventions in previous pandemics (1, 2). Since modern NPIs were adopted over a century ago during the 1918–1919 flu pandemic, much of the public debate has remained unchanged, centering on the efficacy and burdensomeness of NPIs, and their potential for broader effects on morale and economic stability (3, 4).

Public perceptions of NPIs may be an important determinant of compliance (5–9). Moreover, the intensity of public scrutiny surrounding COVID-19 NPI adoption may further heighten the importance of public buy-in in developing meaningful and robust public health solutions (10–13). Public adoption of NPIs may also be region-specific, as one study demonstrated significant variation in willingness to use NPIs in response to Severe Acute Respiratory Syndrome (SARS) outbreaks that may be of cultural origin (14). Others have explored the efficacy of various NPIs in response to a range of emerging infectious diseases, including swine flu, and Ebola (15, 16). Pandemic responsiveness is contingent on individuals eschewing their normal daily behaviors; thus, a small number of refusers may drive—and social media may further exacerbate—such behaviors. Some have suggested that NPI adherence is improved with improved communication; that is, NPI non-adherence is the result of a knowledge gap (17–25). Yet data from behavioral research suggests that non-compliance with expert recommendations is sometimes *not* a result of a lack of knowledge *per se* (26–31).

Understanding whether outcome expectations (a perception of *efficacy*) affect NPI adherence is critical; if there is a knowledge gap in appreciating that NPIs are effective, it could be addressed through outreach efforts. Conversely, if NPI non-adherence is a function of process expectations (concerns that performing NPIs is too *onerous*), then outreach efforts could be focused on mitigating these perceptions rather than highlighting the potential to reduce disease spread.

We therefore sought to understand public attitudes and beliefs regarding various NPIs and self-reported adoption of NPIs, and to explore associations between NPI performance and the baseline characteristics of respondents. These data may help inform public health efforts, as a better understanding of the drivers of refusal to engage in NPIs will help tailor messaging appropriately and ideally increase the chances of encouraging behavioral changes that may ultimately result in reduced disease transmission.

## Methods

We developed a cross-sectional online survey of the general US population after iterative pilot testing. This study was deemed exempt by the Ascension Health institutional review board. The survey was prepared on the Qualtrics platform (Qualtrics Corp, Provo, Utah) and distributed to a representative US sample stratified by age, sex, and race, through Prolific Academic (Oxford, United Kingdom), a platform for academic survey research (32). Prolific Academic maintains a database of over 100,000 potential survey respondents, approximately one-third of whom reside in the US (10, 33). By stratifying on age, race, and sex, the company is able to provide a representative sample of the US general population. Respondents were rewarded with a small payment (<US$1). Subjects provided consent and were allowed to terminate the survey at any time, and all responses were confidential. Sample size calculations were performed a priori for a separate study using this dataset to study mental health outcomes in the COVID-19 pandemic (34); *post hoc* sample size calculations demonstrated that a sample size of 1,000 respondents would yield 95% confidence intervals with a clinically meaningful margin of error of ± 3.1% when taking the entire adult population of the US as our population of interest.

Baseline responses to survey questions were recorded, and demographic information was self-reported by respondents. Responses to a range of questions regarding attitudes to the COVID-19 pandemic, fears, worries, and NPI beliefs and actions were collected using Likert scales. These questions were developed and refined *de novo* using iterative online focus group testing. Key questions addressed included NPI performance/ adherence over the past week (with Likert-type responses), beliefs regarding the efficacy of individual NPIs in slowing the spread of COVID-19 (with Likert-type response options), and stated beliefs regarding whether adherence to NPIs would reduce the personal likelihood of contracting COVID-19 (with Likert-type responses).

*T*-tests and chi-squared tests were seen as appropriate for baseline continuous and categorical variables. Subgroup comparisons of non-normally distributed data were performed using the Kruskal Wallis test. Univariate logistic regression odds ratios of association were assessed between the dependent variable of NPI adherence, defined as those who engaged, on average, in each NPI always or most of the time, and baseline characteristics and attitudes. Statistical analyses were performed using Stata 13 for Mac (College Station, Texas).

## Results

Of the 1,020 subjects who were recruited, 1,005 finished the survey, yielding a completion rate of 98.5%. The mean (SD) age of respondents was 45 (16), and 494 (48.8%) of the respondents were male; baseline respondent characteristics are outlined in Table 1. Surveys were returned between March 29 and March 31, 2020; by this time, the federal government had already issued nationwide social distancing guidelines and 35 states had already enacted stay-at-home orders of some sort.

**Table 1:** Demographic and baseline characteristics of respondents, overall and by social distancing adherence, and whether respondents were under a government requirement to remain at home.

		**Social distancing**		**Required by the government to remain at home**	
**Characteristic**	**Total**	**Always**	**Not Always**	**Yes**	**No**
Overall	1,005 (100)	736 (72.2)	284 (27.8)	681 (66. 8)	389 (33.2)
**Sex****^†^**					
Men	494 (48.8)	347 (47.2)	147 (53.3)	310 (46.1)	184 (54.3)
Women	518 (51.2)	389 (52.9)	129 (46.7)	363 (53.9)	155 (45.7)
**Age, y**					
18–30	250 (24.5)	171 (23.2)	79 (27.8)	165 (24.2)	85 (25.1)
31–40	204 (20.0)	152 (20.7)	52 (18.3)	139 (20.4)	65 (19.2)
41–50	146 (14.3)	108 (14.7)	38 (13.4)	100 (14.7)	46 (13.6)
51–60	198 (198.4)	151 (20.5)	47 (16.6)	130 (19.1)	68 (20.1)
>60	222 (21.8)	154 (20.9)	68 (23.9)	147 (21.6)	75 (22.1)
Education level*					
< High school	11 (1.1)	4 (0.5)	7 (2.6)	8 (1.2)	3 (0.9)
High school	117 (11.7)	77 (10.5)	40 (15.0)	67 (10.1)	50 (14.8)
Some college	228 (22.8)	166 (22.6)	62 (23.2)	149 (22.4)	79 (23.4)
Associates	103 (10.3)	72 (9.8)	31 (11.6)	66 (9.9)	37 (11.0)
Bachelor's	358 (35.7)	272 (37.0)	86 (32.2)	246 (37.0)	112 (33.2)
Graduate	185 (18.5)	144 (19.6)	41 (15.4)	129 (19.4)	56 (16.6)
**Employment status**					
Full time	461 (45.2)	339 (46.1)	122 (43.0)	303 (44.5)	158 (46.6)
Part time	170 (16.7)	118 (16.0)	52 (18.3)	115 (16.9)	55 (16.2)
Not employed	389 (38.1)	279 (37.9)	110 (38.7)	263 (38.6)	127 (37.2)
**Religious**					
Yes	387 (37.9)	279 (37.9)	108 (38.0)	252 (37.0)	135 (39.8)
No	543 (53.2)	391 (53.1)	152 (53.5)	361 (53.0)	182 (53.7)
Ambivalent	90 (8.8)	66 (9.0)	24 (8.5)	68 (10.0)	22 (6.5)
**Income**					
< $10,000	167 (16.4)	121 (16.4)	46 (16.2)	115 (16.9)	52 (15.3)
$10,000–$30,000	234 (22.9)	169 (23.0)	65 (22.9)	154 (22.6)	80 (23.6)
$30,001–$50,000	220 (21.6)	155 (21.1)	65 (22.9)	137 (20.1)	83 (24.5)
$50,001–$80,000	201 (19.7)	151 (20.5)	50 (17.6)	131 (19.2)	70 (20.7)
$80,001–$100,000	63 (6.2)	50 (6.8)	13 (4.6)	42 (6.2)	21 (6.2)
$100,001–$150,000	91 (8.9)	56 (7.6)	35 (12.3)	71 (10.4)	20 (5.9)
> $150,000	44 (4.3)	34 (4.6)	10 (3.5)	31 (4.6)	13 (3.8)
**Location****^†^**					
Urban	743 (72.8)	543 (73.8)	200 (70.4)	517 (75.9)	226 (66.7)
Rural	277 (27.2)	193 (26.2)	84 (29.6)	164 (24.1)	113 (33.3)

*All values are listed as number (%)*.

* *p < 0.05 by chi squared test (social distancing)*.

† *p < 0.05 by chi squared test (required to stay at home)*.

More than 90% of subjects reported using several common NPIs either all or most of the time (Table 2). Respondents were most uncertain regarding the efficacy of mask and eye protection use, with 30.6 and 22.1%, respectively, unsure whether their use would slow disease spread. Overall, 37% (34.0, 39.9) of respondents felt that NPIs in general were difficult to perform (or inconvenient), while only 0.9% (0.3, 1.5) of respondents believed that NPIs in general would *not* reduce their personal risk of illness.

**Table 2:** Non-pharmaceutical intervention performance frequency and belief level.

	**Performed in last week, frequency**, ***n*** **(%)**					**Slows the Spread of COVID-19, level of agreement**, ***n*** **(%)**				
**NPI**	**Always**	**Most of the time**	**Sometimes**	**Rarely**	**Never**	**Completely agree**	**Agree**	**Unsure**	**Disagree**	**Disagree completely**
Hand washing	776 (77.2)	188 (18.7)	29 (2.9)	9 (0.9)	3 (0.3)	871 (86.7)	124 (12.3)	9 (0.9)	0 (0)	1 (0.1)
Hand sanitizer	355 (35.6)	192 (19.3)	222 (22.3)	95 (9.5)	132 (13.3)	722 (71.9)	224 (22.3)	45 (4.5)	7 (0.7)	6 (0.6)
Avoiding handshakes	875 (87.2)	67 (6.7)	42 (4.2)	9 (0.9)	10 (1.0)	819 (81.9)	164 (16.4)	13 (1.3)	2 (0.2)	2 (0.2)
Tissue/ elbow sneeze	749 (74.6)	170 (16.9)	50 (5.0)	19 (1.9)	16 (1.6)	793 (78.9)	189 (18.9)	20 (2.0)	2 (0.2)	1 (0.1)
Avoiding face touching	247 (24.6)	356 (35.4)	282 (28.1)	86 (8.6)	34 (3.4)	748 (74.6)	207 (20.7)	42 (4.2)	5 (0.5)	1 (0.1)
Disinfecting surfaces	347 (34.7)	293 (29.3)	242 (24.2)	67 (6.7)	52 (5.2)	745 (74.2)	223 (22.2)	28 (2.8)	5 (0.5)	3 (0.3)
Wearing mask	71 (7.1)	40 (4.0)	95 (9.5)	109 (10.9)	687 (68.6)	420 (41.9)	234 (23.4)	221 (22.1)	89 (8.9)	38 (3.8)
Wearing eye protection	77 (7.7)	45 (4.5)	65 (6.5)	102 (10.2)	709 (71.0)	360 (35.9)	187 (18.6)	307 (30.6)	106 (10.6)	43 (4.3)
Social distancing	736 (73.3)	215 (21.4)	35 (3.5)	12 (1.2)	6 (0.6)	856 (85.9)	123 (12.3)	12 (1.2)	3 (0.3)	3 (0.3)
Avoiding travel	767 (76.6)	171 (17.1)	44 (4.4)	7 (0.7)	12 (1.2)	835 (83.1)	147 (14.6)	19 (1.9)	1 (0.1)	3 (0.3)
Required to stay at home/ quarantine	582 (58.0)	318 (31.7)	64 (6.4)	22 (2.2)	18 (1.8)	846 (84.4)	135 (13.5)	18 (1.8)	0 (0)	4 (0.4)

*All performance-belief pairs were associated significantly (p < 0.001)*.

On univariate logistic regression analyses, NPI adherence was associated with a belief that NPIs would reduce personal risk of developing COVID-19 [OR 3.06, 95% CI [1.25, 7.48], *p* = 0.014] and with a belief that the NPIs were *not* difficult to perform [OR 1.79, 95% CI [1.38, 2.31], *p* < 0.0001]. Adherence was also associated with self-described religiosity [OR 1.85, 95% CI [1.42, 2.39], *p* < 0.0001]; full-time employment [OR 1.35, 95% CI [1.02, 1.78], *p* = 0.035]; worry regarding a family member contracting COVID-19 [OR 1.47, 95% CI [1.11, 1.93], *p* = 0.007]; and belief that the media was *not* exaggerating the severity of the pandemic [OR 1.44, 95% CI [1.09, 1.91], *p* = 0.012].

## Discussion

Most respondents stated that they were performing key NPIs, such as hand washing and social distancing, on a consistent basis, and the majority of respondents agreed that NPIs are effective in slowing the spread of COVID-19. Mask wearing and eye protection adherence and perceived efficacy lag behind other NPIs; this may be due to messaging, since at the time the survey was performed no recommendations were in place to encourage mask or face protection by the general public in the US. While some have questioned the effectiveness of school closures (35), it is important to maintain consistent messaging for the general public, particularly since the scientific consensus is that NPIs are effective overall (2, 5, 6). This is particularly important since beyond belief in efficacy, emotional appeals may be important in encouraging appropriate behaviors (36). Not surprisingly, those who believe that NPI use is not at all difficult to engage in/inconvenient are more likely to engage in NPI use, as are those that believe in the efficacy of NPIs in reducing personal risk of COVID-19 infection. Our single study incudes approximately the same number of subjects as all 16 studies included in a recent systematic review of influenza pandemic beliefs (37).

Limitations of this survey-based study include: generalizability, mitigated in part by the stratified sampling and large survey panel design; response and social desirability biases, the latter reduced by the anonymous nature of the survey; and the inability to draw causal inferences from a cross-sectional investigation.

These data highlight potential targets for public health efforts: respondents were compliant with straightforward, familiar, and heavily-encouraged NPI recommendations such as hand-washing; more onerous approaches, such as avoiding face touching, disinfecting surfaces, and wearing masks or goggles, were performed less frequently. These findings are consistent with previous research on NPIs for pandemic influenza (6). Changes in CDC recommendations for mask/ face coverings may impact these behaviors in the future.

Given these findings, several steps could be considered to encourage future NPI adoption. First, make it clear: consistent messaging from the government and other community leaders on the effectiveness of NPIs may lower the threshold for community buy-in. The public should understand that NPIs have an effect on their personal risk of contracting COVID-19, as well as the risk of others becoming infected. Second, make it easy: compliance with NPIs should not be onerous. This applies to both practical aspects of NPI adherence—masks and hand sanitizer must be easily and, ideally, freely available—as well as to the social underpinnings of NPI adherence. One study previously demonstrated that the public in countries where wearing masks is de rigueur are more likely to engage in mask wearing in response to a pandemic (14). Thus, highlighting that mask-wearing (and other NPIs) are socially expected, rather than socially awkward, may be helpful.

An improved understanding of the drivers of refusal to engage in NPIs may help tailor messaging and increase the chances of eliciting behavioral change. NPI non-adherence is associated with both outcome expectations (NPIs are *effective*) and process expectations (NPIs are *inconvenient*). These findings have important implications for designing public health outreach efforts, where the feasibility, as well as the effectiveness, of NPIs should be stressed.

## Data Availability Statement

The raw data supporting the conclusions of this article will be made available by the authors, without undue reservation.

## Ethics Statement

The studies involving human participants were reviewed and approved by Ascension Health IRB. The ethics committee waived the requirement of written informed consent for participation.

## Author Contributions

JK and BK: study conception, statistical analysis, survey development, and manuscript preparation. JK: oversight. All authors contributed to the article and approved the submitted version.

## Conflict of Interest

The authors declare that the research was conducted in the absence of any commercial or financial relationships that could be construed as a potential conflict of interest.
